# Detecting microstructural alterations of cerebral white matter associated with breast cancer and chemotherapy revealed by generalized q-sampling MRI

**DOI:** 10.3389/fpsyt.2023.1161246

**Published:** 2023-06-09

**Authors:** Vincent Chin-Hung Chen, Wei Chuang, Chien-Wei Chen, Yuan-Hsiung Tsai, Roger S. McIntyre, Jun-Cheng Weng

**Affiliations:** ^1^School of Medicine, Chang Gung University, Taoyuan, Taiwan; ^2^Department of Psychiatry, Chang Gung Memorial Hospital, Chiayi, Taiwan; ^3^Department of Medical Imaging and Radiological Sciences, Department of Artificial Intelligence, Chang Gung University, Taoyuan, Taiwan; ^4^Department of Diagnostic Radiology, Chang Gung Memorial Hospital, Chiayi, Taiwan; ^5^Mood Disorder Psychopharmacology Unit, Department of Psychiatry, University Health Network, University of Toronto, Toronto, ON, Canada; ^6^Institute of Medical Science, University of Toronto, Toronto, ON, Canada; ^7^Departments of Psychiatry and Pharmacology, University of Toronto, Toronto, ON, Canada; ^8^Medical Imaging Research Center, Institute for Radiological Research, Chang Gung University, Taoyuan, Taiwan

**Keywords:** breast cancer, chemotherapy, psychiatric comorbidity, generalized q-sampling imaging, graph theoretical analysis

## Abstract

**Objective:**

Previous studies have discussed the impact of chemotherapy on the brain microstructure. There is no evidence of the impact regarding cancer-related psychiatric comorbidity on cancer survivors. We aimed to evaluate the impact of both chemotherapy and mental health problem on brain microstructural alterations and consequent cognitive dysfunction in breast cancer survivors.

**Methods:**

In this cross-sectional study conducted in a tertiary center, data from 125 female breast cancer survivors who had not received chemotherapy (BB = 65; 49.86 ± 8.23 years) and had received chemotherapy (BA = 60; 49.82 ± 7.89 years) as well as from 71 age-matched healthy controls (47.18 ± 8.08 years) was collected. Chemotherapeutic agents used were docetaxel and epirubicin. We used neuropsychological testing and questionnaire to evaluate psychiatric comorbidity, cognitive dysfunction as well as generalized sampling imaging (GQI) and graph theoretical analysis (GTA) to detect microstructural alterations in the brain.

**Findings:**

Cross-comparison between groups revealed that neurotoxicity caused by chemotherapy and cancer-related psychiatric comorbidity may affect the corpus callosum and middle frontal gyrus. In addition, GQI indices were correlated with the testing scores of cognitive function, quality of life, anxiety, and depression. Furthermore, weaker connections between brain regions and lower segregated ability were found in the post-treatment group.

**Conclusion:**

This study suggests that chemotherapy and cancer-related mental health problem both play an important role in the development of white matter alterations and cognitive dysfunction.

## Introduction

In the past few decades, breast cancer-related mortality rates have declined by 28–65% because of early detection by mammography and the use of adjuvant therapy to prevent tumor recurrence (1). Many studies have reported cognitive impairments following adjuvant chemotherapy in breast cancer survivors (2–5). In addition, cancer-related post-traumatic stress symptom (PTSS) has been widely reported by cancer survivors (6). Prior studies had demonstrated cognitive changes in patients with breast cancer before they received chemotherapy, indicating that psychological distress (due to the general impact of cancer diagnosis and treatment) may be a possible contributor to cognitive dysfunction (7–9).

Assessment of the impact of cancer treatment on cognitive ability usually involves subjective/ objective questionnaire assessment and neuropsychological testing. However, the cancer-related cognitive deficits and underlying neurological basis are still unresolved (10, 11). These deficits can result from the primary tumor, the treatment received, or both, and can affect multiple cognitive domains, such as memory, attention, executive function, and processing speed. Furthermore, these cognitive impairments can have a significant impact on quality of life, social functioning, and vocational outcomes in cancer survivors. The long-term effects of altered brain network organization caused by chemotherapy are still being studied, but some possible effects include (1) cognitive changes (such as memory deficits, attention problems, and difficulties with executive functions), (2) emotional changes (emotional processing leads to mood changes and increased risk of depression and anxiety), and (3) quality of life (including their ability to perform daily activities and engage in social interactions) (2–5, 10, 11).

One specific problem that needs to be addressed is the identification of reliable imaging markers that can accurately predict the risk of cognitive decline in cancer patients undergoing treatment. Recently, neuroimaging has been used as one of the objective methods for examining cancer-related effects to understand the underlying processes leading to cognitive changes. Diffusion tensor imaging (DTI) is a diffusion magnetic resonance imaging (MRI) technique that can provide white matter (WM) microstructure information, and it has been widely used to detect WM alteration in cancer survivors (12). However, many scholars have found that DTI-based reconstruction of intracranial nerve fibers has several limitations. First, DTI-based reconstruction failed to achieve satisfactory visualization of the brainstem and revealed missing, incomplete, and broken fibers (13). Second, DTI cannot help visualize fibers in tumors or regions of edema (14). To overcome the limitations of DTI, the present study used generalized q-sampling imaging (GQI) as an imaging modality to detect alterations in brain microstructures. GQI is an advanced diffusion MRI method and has been used to determine how topological alterations in the brain network are affected by mental illnesses such as Alzheimer’s disease, multiple sclerosis, and epilepsy (15). GQI can reconstruct crossing fibers more completely, consistently, and accurately than DTI (16).

The Human brain is such a complex structure that people devote time and effort endeavor to uncover it. The human brain connectome is a comprehensive structural or functional description of the connectivity patterns between different regions of the brain, and gives insights into the brain connections including the distribution and strength of neural pathways, as well as the property of communication. One of the well-known methods of modeling brain connectome is graph theory (17). People reconstruct brain graphs by defining specific regions of the brain as nodes (points) and structural or functional interconnections between regions as edges (lines). Subsequently, topological properties can be measured. Several studies have shown that by combining the performance of topological properties obtained from brain graphs, the human brain is a small-world architecture. It means the brain works complicated with both segregated and integrated functions. On that basis, we could evaluate the differences in brain networks on any topic (18, 19).

Although previous studies have discussed the impact of chemotherapy on brain microstructure (20–25), including dorsal attention network (DAN) and default mode network (DMN), there is still no evidence for the impact of cancer-related psychiatric comorbidity on brain structure of cancer survivors. This study is among the first to evaluate their combined impact. We hypothesized that the neurotoxicity of chemotherapy and cancer-related stress may cause both brain microstructural alterations and cognitive dysfunction in breast cancer survivors. By using GQI and graph theoretical analysis, this study attempted to investigate the impact of chemotherapy and cancer-related mental health problem on brain microstructure and to characterize whole-brain connectivity in breast cancer survivors.

## Materials and methods

### Participants

This study was approved by the Institutional Review Board of Chang Gung Memorial Hospital, Chiayi, Taiwan. (No. 104-5082B, 201700256B0, and 201702027B0). All participants provided informed written consent, and all research was performed in accordance with relevant guidelines and regulations. The cross-sectional study included 65 women with a history of breast cancer who were scheduled to receive chemotherapy (BB), 60 women with a history of BC who had completed their chemotherapy (BA), and 71 age-matched no-cancer controls (HC). Most diffusion MRI studies on breast cancer had focused on the short-term and ultra-long-term effects of chemotherapy (12, 24, 26). To address the gap in research, this study investigated the effects of chemotherapy in patients with breast cancer, 3–12 months after the completion of chemotherapy. Inclusion criteria were as follows: sex, women; age, 20–70 years; diagnosis, by histological confirmation of primary breast cancer; and treatment status, complete. Exclusion criteria were as follows: terminal stage (life expectancy of <1 year); history of presence of any cancer other than breast cancer; undergoing radiation therapy; brain metastasis or other brain lesions, pregnancy or breastfeeding, contraindication for MRI scans, and a history of neurological disorders, severe mental disorders or substance-use disorder. Exclusion criteria for healthy controls were as follows: history of cancer, any history of neurological disorders, severe mental disorders or substance-use disorder, pregnancy or breastfeeding, and contraindications for MRI scans.

### Neuropsychological testing and subjective evaluation

All participants were evaluated by a trained psychotherapist using the structured diagnostic interview of the fifth edition of the Mini-International Neuropsychiatric Interview (27). Neuropsychological tests used in this study was the Mini-Mental State Examination (MMSE). The MMSE has been extensively used to assess the cognitive function and was adopted as a screening tool to assess cognitive dysfunction in cancer survivors and to monitor changes in cognitive status resulting from treatments; higher scores indicate higher cognitive function (28). The subjective (questionnaire) evaluations for psychiatric comorbidity encompassing Hospital Anxiety and Depression Scale (HADS), the Patient Health Questionnaire-9 (PHQ-9), the Cognitive and Affective Mindfulness Scale-Revised (CAMS-R), Impact of Event Scale-Revised (IES-R), and the Functional Assessment of Cancer Therapy–Cognitive Function (FACT-Cog) were performed in this study. The HADS/PHQ-9 are used to determine the degree of anxiety and depression; higher scores indicate that a person is experiencing a higher degree of anxiety or depression (29). The CAMS-R is designed to capture a broad conceptualization of mindfulness with language that is not specific to any particular type of meditation training; higher scores reflect greater qualities related to mindfulness (30). The IES-R involves a self-reported assessment of PTSS caused by traumatic events over the past 7 days; higher scores reflect a greater distress or bothering (31). The FACT-Cog questionnaire, which includes four subscales, that is (1), perceived cognitive impairments (2), comments from others (3), perceived cognitive abilities, and (4) impact on the quality of life, was developed to assess perceived cognitive function in cancer survivors; higher scores reflect lesser cognitive deficit (32, 33).

### Statistical analysis

Variables are expressed as means ± standard deviations (SDs) for normally distributed data, and medians and interquartile ranges (IQRs) for data that are not normally distributed. The Kolmogorov–Smirnov Test was used for testing normality of variables. The Kruskal-Wallis Test was used for testing differences between groups. For image analysis, voxel-based analysis (VBA) and graph theoretical analysis (GTA) were both performed, and the critical alpha value was set as 0.05 corrected by false discovery rate (FDR) for significance level of all statistical analyses.

### Magnetic resonance imaging data acquisition

All participants underwent diffusion MRI, which was performed using a 3-Tesla MRI scanner (Magnetom Verio, Siemens, Erlangen, Germany) with parameter repetition time = 8,943 ms; echo time = 115 ms; field of view = 250 mm^2^; matrix = 128 × 128; slice thickness = 4 mm; b-values = 0, 1,000, 1,500, and 2000 s/mm^2^ in 64 non-collinear directions; number of excitations = 1; and acquisition time = 30 min. The participants were instructed to lie down and remain motionless. Cushions and earmuffs were used to reduce participant motion and scanner noise. All the participants underwent other MRI pulse sequences, including axial T2-weighted imaging (T2WI), T1-weighted imaging (T1WI), fluid-attenuated inversion recovery, and coronal T2WI; additionally, participants with breast cancer underwent postcontrast axial, coronal, and sagittal T1WI, on the basis of which we ruled out the possibility of brain metastasis.

### Image preprocessing and analysis

We used four methods, namely, voxel-based analysis (VBA), multiple regression analysis, graph theoretical analysis (GTA), and network-based statistical (NBS) analysis, to analyze the diffusion MRI data. VBA was first performed by correcting the diffusion images for eddy currents using FSL (FMRIB software library). The spin distribution function was reconstructed using a model-free reconstruction method with DSI Studio (developed by Fang-Cheng (Frank) Yeh). Using this mathematical algorithm, we obtained the diffusion indices of GQI, including generalized fractional anisotropy (GFA), quantitative anisotropy, normalized quantitative anisotropy (NQA), and isotropic value of the orientation distribution function (ISO). Voxel-based comparisons of GQI indices were performed by analysis of covariance (ANCOVA) to examine the group differences among the groups of BB, BA, and HC in Statistical Parametric Mapping (SPM) software. Post-hoc tests were then used for comparison between groups. Age and years of education were taken into account and set as covariates of no interest. The results of significant voxels would be visualized as t-score map and overlapped on GFA/NQA map using xjView toolbox.^1^ Multiple regression analysis was also performed using SPM software to detect correlations between neuropsychological scale scores and the diffusion indices of GQI of all participants. We also used age and years of education as covariates in the multiple regression analysis.

### Network construction and graph theoretical analysis

We reconstructed the pathways of nerve fibers in the brain using the Fiber Assignment with Continuous Tracking (FACT) algorithm with DSI studio. Network edges were established using FACT and the Automated Anatomical Labeling templates, which divided the brain into 90 regions in Montreal Neurological Institute space. The number of virtual fibers, or “edges,” connecting each pair of regions was determined, and 90 × 90 weighted connectivity matrices were identified for each participant. The graph theoretical algorithm was used to determine topological indices, including mean clustering coefficient, gamma, local efficiency, characteristic path length, lambda, and global efficiency. The mean clustering coefficient, gamma, and local efficiency were used for evaluating network segregation, and the characteristic path length, lambda, and global efficiency were used for evaluating network integration. The Area under the Curve (AUC) for each connectivity metric of the topological indices was compared between groups. To identify statistically significant differences in the network topological indices between groups, graph theoretical analysis toolbox was used to execute the two-sample t-test and non-parametric permutation test with 1,000 repetitions.

### Network-based statistical analysis

The Network-Based Statistic (NBS, Melbourne Neuropsychiatry Centre, The University of Melbourne and Melbourne Health, Australia) is a toolbox for testing hypotheses about the connectome of human brain. NBS analysis has been widely used to identify connections and networks comprising the connectome associated with a between-group difference (34). NBS analysis is used to identify any potentially connected structures formed by an appropriately chosen set of supra-threshold links. The topology of any such structure is used to examine its significance. The test statistic (i.e., primary threshold) computed for each pairwise combination is used to construct a set of supra-threshold links. The null distribution of the number of edges was empirically acquired using nonparametric permutation (5,000 permutations) to evaluate the significance of each of the connected edges. Finally, we used the BrainNet viewer (The MathWorks Inc., Natick, MA, United States) to visualize the significant sub-networks revealed by the NBS.

## Results

### Participant demographics and cognitive testing

We recruited a total of 196 participants from Chiayi Chang Gung Memorial Hospital in our study and assigned them to three groups: women with a history of BC who were scheduled to receive chemotherapy (BB, *n* = 65, age = 49.86 ± 8.23 years), women with a history of BC who had completed their chemotherapeutic regimens approximately 7.5 months ago (BA, *n* = 60, age = 49.82 ± 7.89 years) and age-matched no-cancer controls (HC, *n* = 71, age = 47.18 ± 8.08 years). Their age ranged from 29 to 68 years old and had no statistically differenced among groups (*p* = 0.055). Because of a wide variation and significant differences in education year between groups (*p* = 0.044) which were evaluated by Kruskal-Wallis test, we considered age and education year as the covariant factors in statistical analysis to reduce its impact. However, the significance values did not meet the criteria (*α* = 0.05) after adjusting by the Bonferroni correction in post-hoc tests. Stage 0, I, II, III, and IV cancers were noted in 18, 20, 20, 5, and 2 patients in group BB and 0, 11, 33, 12, and 4 patients in group BA, respectively. Neuropsychological test (MMSE) and HADS-anxiety score showed no significant difference between groups. Subjective evaluation (CAMS-R, IESR, and FACT-Cog) scores were lower in group BA than in group BB, but not at a statistically significant level. Participant demographic information and neuropsychological assessment results are presented in Table 1. There was significant difference of PHQ-9 between BB and HC.

**Table 1:** Demographic characteristics in cross-sectional study.

	BB (*n* = 65)	BA (*n* = 60)	HC (*n* = 71)	Kruskal-Wallis test	BB *vs* HC	BA *vs* HC	BB *vs* BA
	Mean/Median/No.	Mean/Median/No.	Mean/Median/No.	*p*	*p* (adjusted by Bonferroni correction)		
Age (years), mean (SD)	49.86 (8.23)	49.82 (7.89)	47.18 (8.08)	0.055			
Education (years), mean (SD)	11.94 (3.87)	11.62 (3.71)	12.98 (3.29)	0.044*	0.134	0.072	1
Days after last cycle of chemotherapy (days), mean (SD)	N/A	231 (149)	N/A				
Breast cancer stage							
0	18	0	N/A				
I	20	11	N/A				
II	20	33	N/A				
III	5	12	N/A				
IV	2	4	N/A				
PHQ9, mean (SD)	4.77 (4.16)	3.92 (3.93)	2.97 (3.00)	0.024*	0.019*	0.64	0.49
HADS-anxiety, mean (SD)	5.05 (4.52)	3.75 (3.88)	3.79 (3.63)	0.097			
MMSE, median (IQR)	29 (3.5)	29 (2.5)	29 (2)	0.961			
CAMS-R, median (IQR)	34 (8)	31 (10)	34 (9.5)	0.741			
IES-R, median (IQR)	20.5 (35.8)	18 (22)	N/A	0.901			
FACT-Cog, median (IQR)							
Perceived cognitive impairments	63 (22.3)	57 (29.5)	65.5 (11.5)	0.250			
Comments from others	16 (0.75)	15 (5)	16 (2)	0.246			
Perceived cognitive abilities	19.5 (7.8)	12 (8.5)	19 (9.3)	0.185			
Impact on quality of life	14 (7.8)	12 (5.5)	15 (3.8)	0.690			

CAMS-R, cognitive and affective mindfulness scale-revised; FACT-Cog, functional assessment of cancer therapy-cognitive function; HADS, hospital anxiety and depression scale; IES-R, impact of event scale-revised; IQR, interquartile range; MMSE, mini-mental state examination; N/A, not applicable; PHQ-9, Patient health questionnaire-9, SD, standard deviation.BB group: Breast cancer women who had not received chemotherapy.

BA group: Breast cancer women who had received chemotherapy.

HC group: Age-matched healthy control women.

*The Kruskal-Wallis Test was used for testing differences between groups. If the difference reached statistical significance (*p* < 0.05).

### Voxel-based analysis

**Figure 1:**
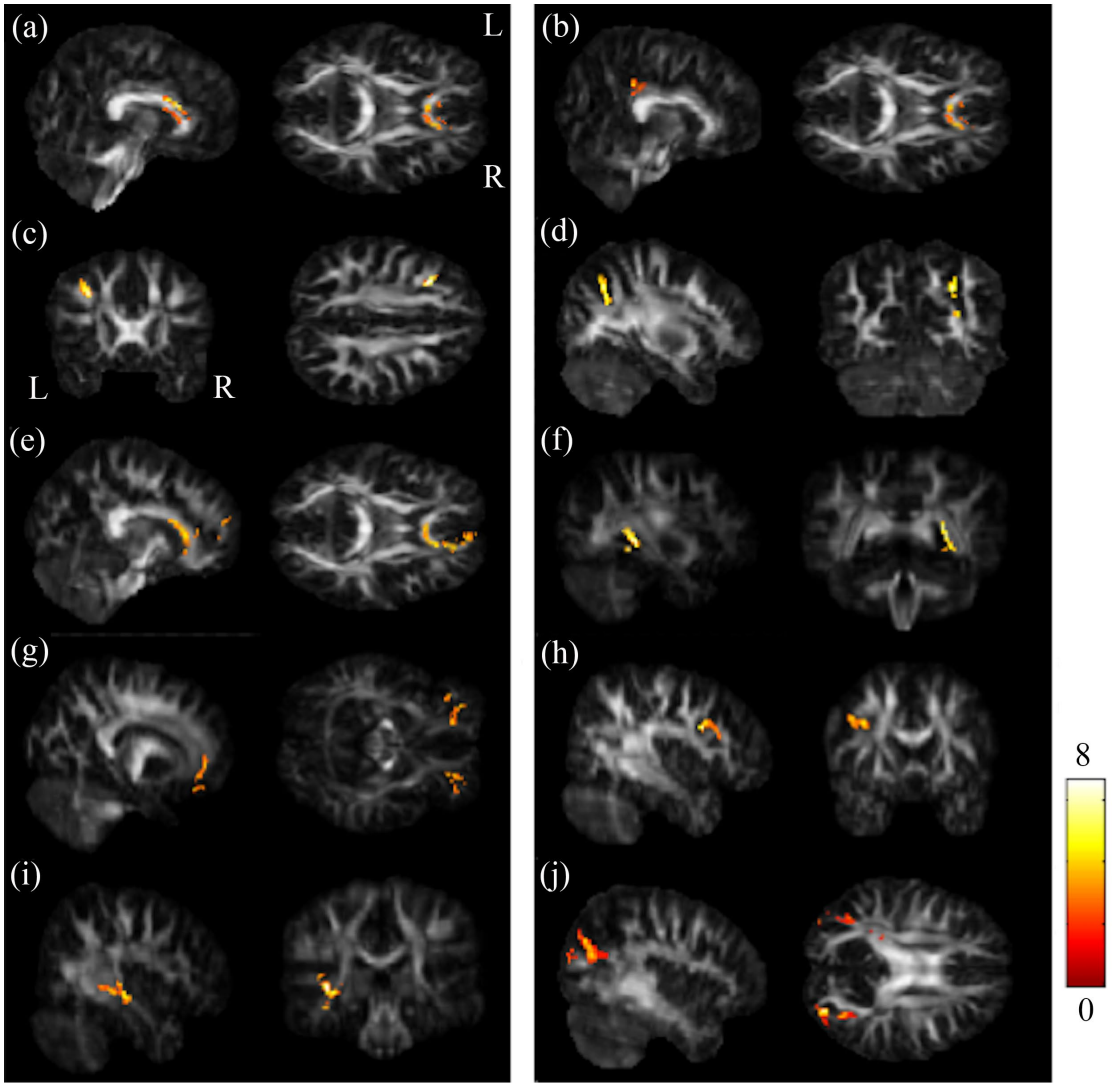
Results of ANCOVA among BB, BA and HC groups. Among BB, BA and HC groups, notable differences were found in GFA in the regions of **(A)** CC, **(B)** l-PCG, **(C)** l-MFG, and **(D)** r-SPG; and significant differences were seen in NQA in the regions of **(E)** CC, **(F)** r-tapetum, **(G)** bi-ORBmidF, **(H)** l-MFG, **(I)** l-ILF, and **(J)** bi-MOG (*α* = 0.05). The color bar represents F-scores. Please look for the abbreviation details in the Appendix.

The ANCOVA demonstrated that the GFA maps had significant differences in the regions of corpus callosum (CC), left posterior cingulate gyrus (PCG), left middle frontal gyrus (MFG) and right superior parietal gyrus (SPG). And the NQA maps showed significant differences in the regions of corpus callosum (CC), right tapetum, bilateral orbital part of middle frontal gyrus (ORBmidF), left middle frontal gyrus (MFG), left inferior longitudinal fasciculus (ILF), and bilateral middle occipital gyrus (MOG) among these three groups (Figure 1).

**Figure 2:**
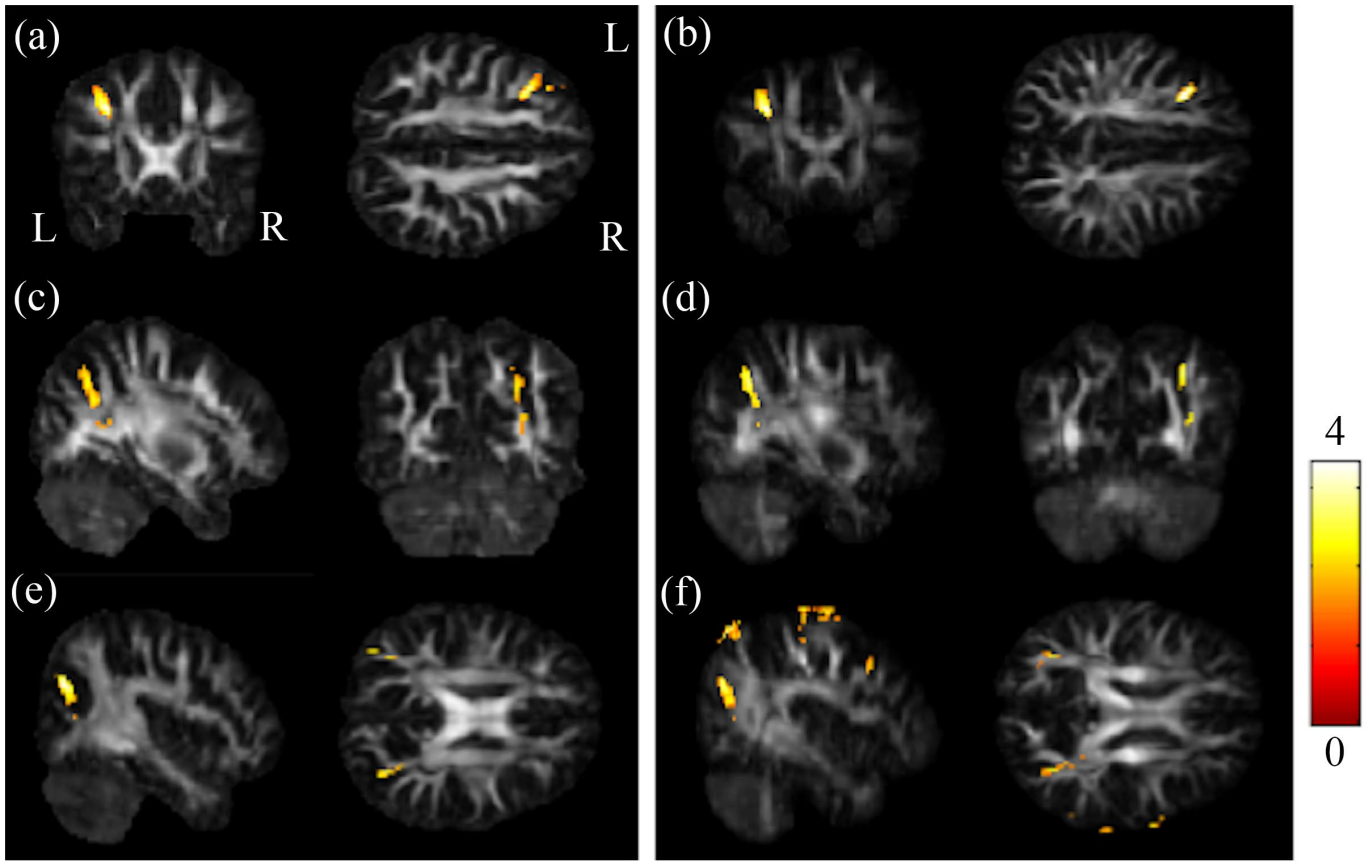
Results of post-hoc t-test between BB and HC groups. Regions showing remarkably reduced GFA/NQA values in pre-chemotherapy patients in **(A,B)** l-MFG, **(C,D)** r-SPG, and **(E,F)** bi-MOG. The left column was the results of GFA. And the right column was the results of NQA (*α* = 0.05). The color bar represents t-scores of t-statistics. Please look for the abbreviation details in the Appendix.

**Figure 3:**
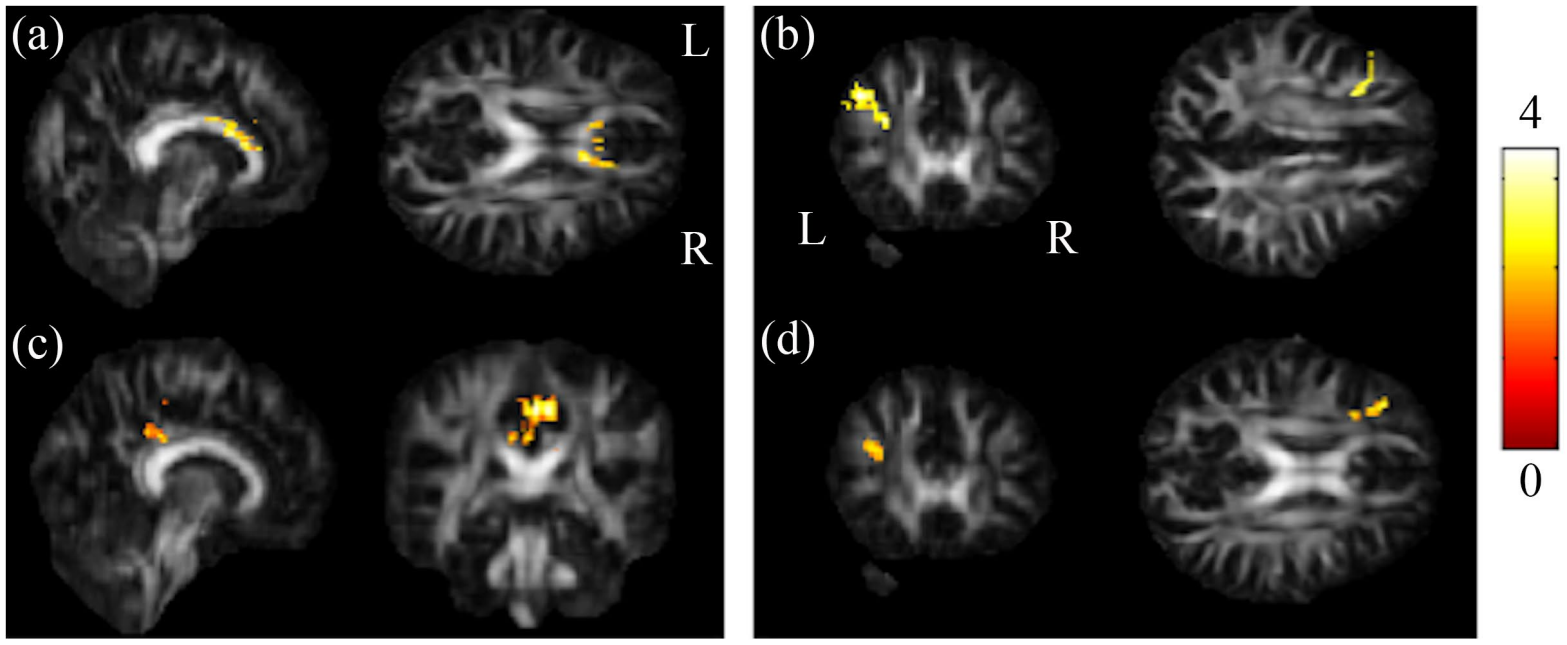
Results of post-hoc t-test showed HC and BB groups larger than BA group. Results of this study demonstrated that the chemotherapy-treated group had significant lower GFA values in **(A)** CC as well as **(B)** l-MFG compared to HC, and reduced GFA values in **(C)** l-PCG as well as **(D)** l-MFG compared to BB (*α* = 0.05). The color bar represents t-scores of t-statistics. Please look for the abbreviation details in the Appendix.

The post-hoc t-test revealed that in the group of BB, both GFA and NQA notably decreased in left MFG, right SPG, and bilateral MOG compared to HC (Figure 2). The GFA of the post-chemotherapy group were significantly lower in the CC and left MFG than HC (Figures 3A,B), and remarkably lower in left PCG and left MFG than the pre-chemotherapy group (Figures 3C,D). All the results above were listed in Supplementary Table S1.

### Multiple regression analysis

**Figure 4:**
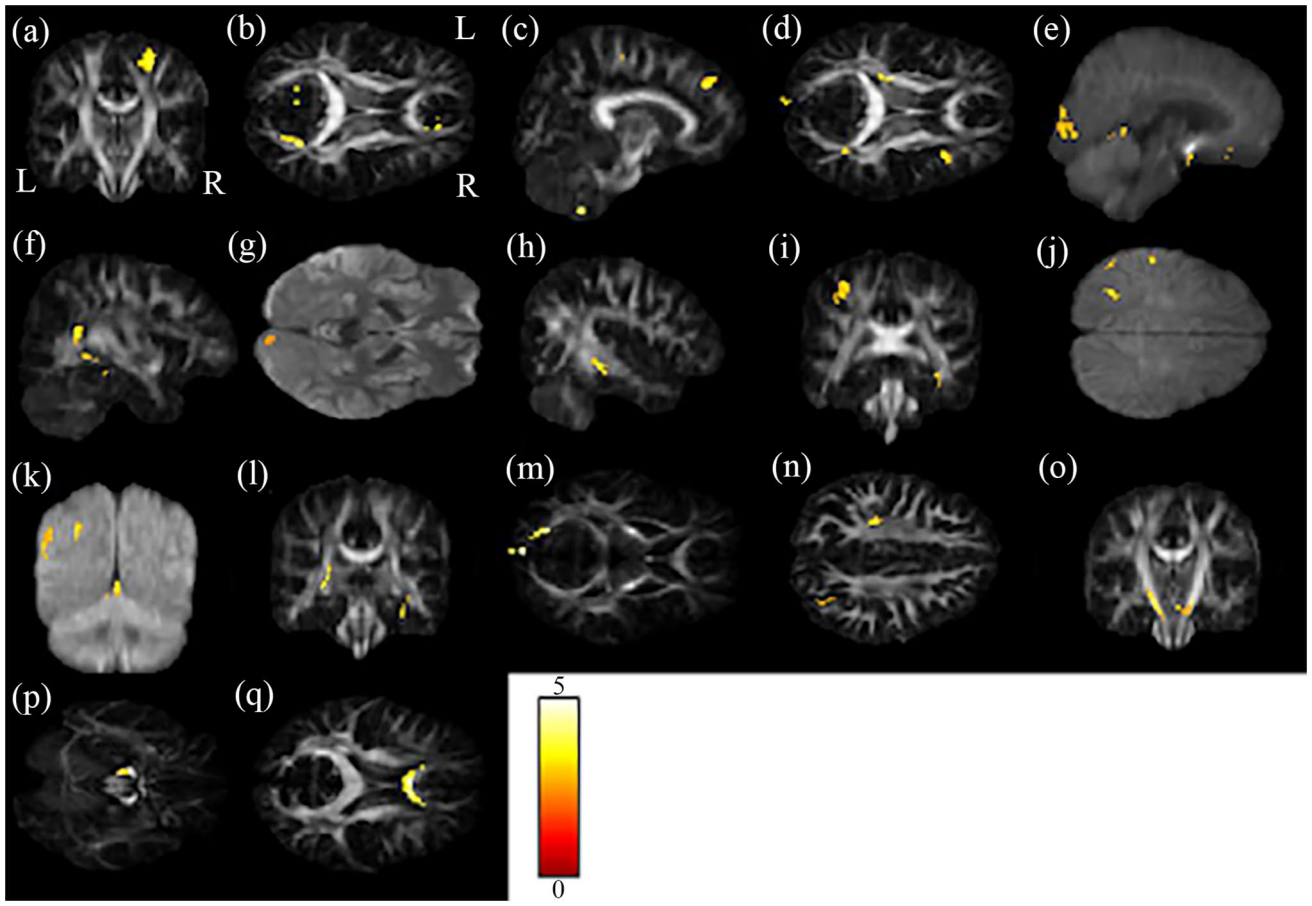
The results of the brain regions included in the multiple regression analysis. The brain regions **(A–Q)** in the figure correspond to the second column of Table 2.

Correlations of scores of cognition test and rating scales (i.e., MMSE, HADS-anxiety, PHQ-9, CAMS-R, IES-R, and FACT-Cog) with changes in GQI indices were evaluated. The results of the multiple regression analysis are shown in Table 2; Figure 4. It is worth mentioning that MMSE and FACT-Cog scores (comments from others, impact on the quality of life) were positively correlated with the GQI indices in some brain regions, including the right precentral blade, right posterior thalamic radiation, right superior frontal blade, and right sagittal stratum (*p* < 0.01 corrected by FDR). On the contrary, HADS-anxiety scores and PHQ-9 were negatively correlated with the GQI indices in some brain regions, including the right precentral blade and body of corpus callosum (*p* < 0.01 corrected by FDR).

**Table 2:** Correlation between neuropsychological assessment scales and GQI indices.

							FACT-Cog			
		MMSE	HADS-anxiety	PGQ-9	CAMS-R	IES-R	Perceived cognitive impairments	Comments from others	Perceived cognitive abilities	Impact on quality of life
Right pre-central blade	a	☆	★	★▲						☆△
Right posterior thalamic radiation	b	☆△								
Right superior frontal blade	c				☆	★	☆	☆△	☆	☆
Right inferior frontal blade	d				☆					
Right cuneus	e					○				
Right posterior corna radiata	f				☆					
Right lingual gyrus	g		○				●	●	●	
Right sagital stratum	h			▲			△	☆△	☆	
Left posterior gyrus	i					★○				
Left precuneus	j					○				
Left inferior parietal lobule	k					○				
Left posterior limb of internal capsule	l				☆					☆
Left occipital blade	m				△					
Left superior longitudinal fasciculus	n						☆			
Bilateral cerebral peduncle	o									☆
Left cerebral peduncle	p									△
Body of corpus callosum	q		★▲	★						

☆ = GFA(+), means that GFA was positively correlated with neuropsychological assessment scales.

★ = GFA(−), means that GFA was negatively correlated with neuropsychological assessment scales.

△ = NQA(+), means that NQA was positively correlated with neuropsychological assessment scales.

▲ = NQA(−), means that NQA was negatively correlated with neuropsychological assessment scales.

○ = ISO(+), means that ISO was positively correlated with neuropsychological assessment scales.

● = ISO(−), means that ISO was negatively correlated with neuropsychological assessment scales.

### Graph theoretical and network-based statistical analyses

**Figure 5:**
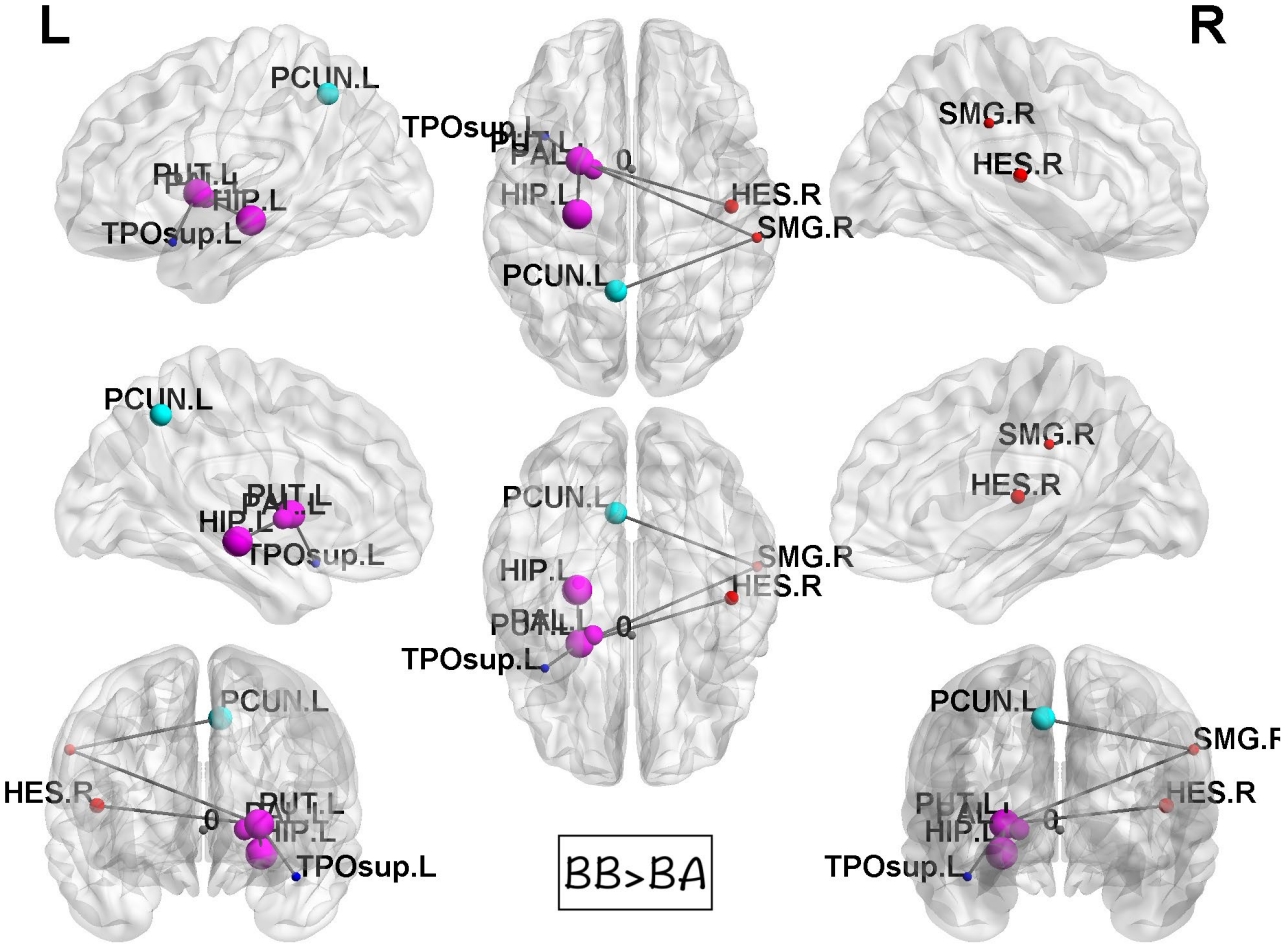
Results of NBS between BB and BA groups. Compared to BA group, the BB group showed statistically significant stronger interconnections in the putamen, hippocampus, precuneus, and temporal lobe.

While computing the topological properties, we integrated the AUC of each parameter by extracting the density range from 0.06 to 0.22. Because fragmentation occurs at network density below 0.05, and the measurements reached a plateau at network density above 0.22. Even though there was no statistical significance could be identified in all the tests. It is worth noting that the architecture still fits the definition of small-world network in these three groups. Among the network-based comparisons, connections in BB were statistically significant higher than BA between the regions of putamen, hippocampus, precuneus, and temporal. The result visualized by using BrainNet Viewer was shown in Figure 5.

## Discussion

Neurotoxicity caused by chemotherapy and cancer-related psychiatric comorbidity are both possible causes of cognitive dysfunction in breast cancer survivors. Although some studies have discussed the impact of chemotherapy on the brain microstructure. However, there is no imaging evidence on the impact of psychiatric comorbidity. This is the first study that used diffusion MRI to investigate the impact of cancer-related psychiatric comorbidity on the brain microstructure. This study provides further evidence indicating that both chemotherapy and cancer-related mental health problem play an important role in the development of white matter alterations and cognitive dysfunction.

By using GQI in combination with graph theoretical analysis, we demonstrated the presence of brain microstructural alterations and cognitive dysfunction in breast cancer survivors. In this study, we found several white matter tract disruptions associated with the dorsal attention network (DAN) in BC patients prior to chemotherapy. Reductions in white matter integrity associated with the default mode network (DMN) in breast cancer patients after receiving chemotherapy. First, VBS analysis revealed the between-group differences of GQI indices in some brain regions, suggesting that neurotoxicity of chemotherapy and cancer-related psychiatric comorbidity may affect corpus callosum (CC) and middle frontal gyrus (MFG). Second, correlations were observed between GQI indices and cognitive testing scores, that is, positive correlation with MMSE and FACT-Cog scores (comments from others, impact on the quality of life) and negative correlation with HADS-anxiety and PHQ-9. Third, graph theoretical analysis showed that each groups of women demonstrated small-world connectomes of WM networks. These findings extend our understanding of the neurophysiologic mechanisms involved, from a network perspective.

### Pretreatment differences

We discovered that the middle frontal gyrus (MFG), superior parietal gyrus (SPG), and middle occipital gyrus (MOG) in the BB group showed significantly lower GQI indices than those in the HC group. The dorsal attention network (DAN) and ventral attention network (VAN) are the two sensory orienting systems in the human brain. The DAN dominated the top-down voluntary control of visuospatial attention, and the VAN involved in the bottom-up system dealt with unexpected stimuli. Both networks are not isolated from each other; they control attention flexibility (35, 36). Many involved regions in the present study were correlated with the DAN.

The literature has indicated that the MFG is associated with literacy and numeracy skills, demanding the cognitive domain of attention. Moreover, the MFG served as the gateway between the DAN and VAN (37, 38). Koenigs et al. identified that the superior parietal is crucial to the manipulation of information in working memory, and damage to this region is associated with deficits on tests involving working memory (39). Additionally, Szczepanski et al. observed several pathways that control spatial attention in the DAN. Different from the pathway between the frontal eye field and intraparietal sulcus, which is viewer-centered, the pathway between the supplementary eye field and superior parietal support viewer- and object-centered representations of attentional properties. The supplementary eye field and superior parietal region (SPG) play an important role in task switching that can flexibly regulate condition-action associations (40). Apart from the frontal and parietal lobes, the occipital lobe was shown to be a component of the DAN, which is related to focusing attention on moving objects. These areas of the DAN are connected by the superior longitudinal fasciculus (SLF) (41, 42). Our results showed disruption of local white matter integrity in the frontal, parietal, and occipital parts of the SLF, consistent with previous findings (12).

These results revealed by GQI were consistent with our team’s previous work on functional MRI (fMRI) (43). We observed that the frontoparietal lobe increased in mean fractional amplitude of low-frequency fluctuation (mfALFF) as well as decreased in mean regional homogeneity (mReHo), and the occipital lobe was lower in mfALFF in the BB group than in the BA and HC groups. Theoretically, structure–function has been shown to have a positive correlation between white matter integrity and human brain activity based on DTI and fMRI assessments (44, 45). In other words, greater white matter integrity enables neurons to recruit more resources simultaneously, and signal transmission is more efficient and rapid (46). This concept was concordant with our results on GQI indices and mReHo. Breast cancer patients without chemotherapy were found to have lower white matter integrity and worse synchronization of spontaneous local brain neuronal activities. Unfortunately, this phenomenon cannot explain the adversarial observations of increased brain activities in the frontoparietal lobe in mfLAFF. A study investigating white matter integrity, brain activation, and cognition suggests that local fiber disintegration contributes to lower cognitive efficiency and higher compensative neural activity in aging and Alzheimer’s disease (47). The same compensatory performance has also been observed in cancer survivors (48, 49).

Several studies have reported that non-CNS cancer patients experience cognitive problems and show brain abnormalities. Whereas van der Willik et al. conducted a population-based cohort study, they found no abnormality in participants before being diagnosed with non-CNS cancer by using brain structural MRI (50). In addition, Deprez et al. demonstrated that no significant difference was found after controlling for depression in breast cancer patients before the start of the chemotherapeutic regimen (12). Menning et al. discovered that cerebral alterations were related to fatigue prior to treatment (51). These findings made certain that the changes before chemotherapy were largely caused by the negative feelings about being diagnosed with breast cancer.

Breast cancer is potentially a traumatic stressor that may lead to psychiatric comorbidity and is associated with psychological distress (52). The literature has proposed that mental health problems are related to altered attention and dysfunction within the dorsal prefrontal network (53, 54). In this study, the PHQ-9 score was significantly higher in the BB group than in HCs; that is, the BB group had a higher level of depression than HCs. The BB group also had a nonsignificantly higher level of anxiety, as revealed by the HADS-A. Combining our results of GQI and fMRI, we suggested that stress disorder was the major cause of DAN alterations in breast cancer patients without chemotherapy.

### Posttreatment differences

The white matter integrity reductions in the corpus callosum (CC), posterior cingulate gyrus (PCG), and middle frontal gyrus (MFG) were found in the BA group compared to the BB or HC group. The corpus callosum is known as the largest white matter bundle in the human brain, communicating between two hemispheres. The anterior part of the CC is responsible for connecting the prefrontal cortex. Several studies have reported the detrimental effect of chemotherapy on CC results in breast cancer patients. In addition, evidence has shown a positive correlation between FA in the CC and processing speed, suggesting that the anterior part of white matter is more susceptible to injuries (12, 55, 56). Raghavan et al. even built a model that can predict mild cognitive impairment by using the FA value of the CC (57).

The PCG is critical in multiple cognitive functions, especially memory. It connects to the hippocampus and carries memory information to other regions. Disrupted PCG was related to mild cognitive impairment. In addition, it is a central part of the default mode network (DMN) (58, 59). In contrast to DAN, the DMN was primarily activated when a person was not focused on the outside world and mind-wandering, supporting self-generated cognition. The functional hubs of the DMN include the posterior cingulate cortex, medial prefrontal cortex, and angular gyrus, which exhibit strong functional coherence with subnetworks of the DMN and allow information to transfer between subsystems. Destruction in hubs might break the stability of the network (60, 61). Previous research in chemotherapy not only found decreased white matter integrity but also detected metabolic changes in the PCG by using magnetic resonance spectroscopy (MRS). They observed that the concentrations of N-acetylaspartate (NAA) and total creatine (tCr) were reduced in the PCG, representing axonal injuries and cell integrity, respectively. The changes in metabolism were also related to memory (62, 63).

The frontal lobe is the largest lobe in the human brain and is involved in a higher level of cognition. Regardless of structural or functional imaging, many studies have reported abnormalities in the frontal lobe in breast cancer patients receiving chemotherapy (24, 48, 64–66). Integrating previous research, Chen et al. suggested that the frontal and temporal lobes are the most sensitive regions to chemotherapy (67). Our results in the MFG had been observed to have correlations with the posterior cingulate cortex and hippocampus, which is part of the DMN (68). The same compensatory hyperactivation in the frontoparietal lobe was also found in our previous study in fMRI data (43).

### Chemotherapy and cognitive alterations

Prior neuroimaging studies have shown that cognitive impairment results in subtle and diffuse brain damage (20–23). Damage to any part of the WM connection can lead to changes in cognitive performance (69). The effects of chemotherapy may lead to deficits in behavior and neuropsychological performance (70). Wefel et al. reviewed 53 published cross-sectional and prospective neuropsychological studies that provided comprehensive evidence on chemotherapy-related cognitive dysfunction in patients with breast cancer and found that attention, memory, processing speed, and executive function were the most commonly affected cognitive domains (71). These effects appear to be more pronounced in the short term; however, Koppelmans et al. revealed that breast cancer survivors exhibited a poorer performance in neuropsychological tests than healthy controls did, even 20 years after they had undergone chemotherapy. Therefore, the effects of chemotherapy on the brain can be long lasting (26). In addition to chemotherapy, Hermelink et al. revealed that cancer-related psychiatric comorbidity may also lead to cognitive dysfunction in patients with breast cancer (6), suggesting that not only chemotherapy but also psychological factors play an important role in breast cancer-related cognitive disorders (6–9). In our study, we did not find any significant differences between groups in terms of neuropsychological assessments. However, neuroimaging analysis showed that there were differences in microstructural alterations between groups, which corroborated the results of the neuropsychological assessments.

The mechanism of development of cognitive dysfunction after chemotherapy remains unclear. There are several potential causes for WM microstructure disruption and cognitive function decline after chemotherapy, notably the direct toxicity of chemotherapeutic drugs against the WM (20, 72). Neurotoxicity is a major adverse reaction to docetaxel; however, docetaxel and epirubicin do not easily cross the blood–brain barrier (72). Fifty-one studies reported the neurological, hematological, and gastrointestinal toxicity associated with docetaxel in adult patients with solid tumors (73). In addition, epirubicin-induced toxicity causes anemia, fever, and neurotoxicity (74). Deprez et al. found that post-chemotherapy patients with breast cancer demonstrated lower fractional anisotropy in the frontal and temporal WM tracts than the healthy controls did (12). Hosseini et al. found that, after chemotherapy, regional connectivity in the frontotemporal region as well as global network organization and integration were lower in breast cancer survivors than in healthy female controls (75). Our results were consistent with those of previous studies.

Several recent clinical and MRI studies have suggested that cognitive alterations, including compromised attention function, can exist in patients with breast cancer before chemotherapy, which may be a result of psychiatric comorbidity (6–9). The mental health problem affect more than 80% of patients with breast cancer after the disease is diagnosed, even before any treatment is begun, and these symptoms could last for more than 1 year (76). Prior research has demonstrated the presence of a smaller hippocampus, amygdala, and prefrontal cortex in individuals with psychiatric comorbidity and a smaller frontal, temporal lobe as well as decreased cognitive function in individuals after chemotherapy (77–80). A functional MRI (fMRI) study found a higher bilateral brain activation in high-demand task conditions with recruitment of additional components of attention/working memory circuitry in prechemotherapy patients with breast cancer than in healthy controls (9). In addition, a positron-emission tomography study revealed that metabolic network in the frontal and temporal gyri was lower in prechemotherapy patients with breast cancer than in healthy controls (81). In our study, GQI results showed that cancer-related psychiatric comorbidity may affect the body of corpus callosum, left inferior frontal gyrus, and left inferior temporal gyrus. Our study used GQI indices for graphical theoretical analysis to investigate brain structural alterations in cancer-related trauma survivors. Notably, we also found correlations between GQI indices and cognitive testing scores, that is positive correlations with MMSE and FACT-Cog scores (comments from others, impact on quality of life) and negative correlations with HADS-anxiety and PHQ-9, which indicated that the alterations in WM connectivity may be associated with psychological distress in breast cancer survivors.

### Chemotherapy and brain connectome

Graphical theoretical analysis (GTA) techniques have become increasingly popular in brain connectivity analysis. The advantages includes (1) GTA provides a holistic view of the brain and allows researchers to visualize and analyze the brain as a network, which provides a more comprehensive understanding of the brain than looking at individual regions in isolation. (2) GTA can capture complex relationships between brain regions, such as the presence of hubs, clusters, and modules. This provides insights into the organization and communication patterns of the brain. However, the disadvantages includes (1) GTA does not provide a clear understanding of causality. While it can identify associations between brain regions, it cannot determine whether one region is causing changes in another or vice versa. (2) GTA ignores regional properties and focuses on the overall network properties of the brain and can ignore important regional properties, such as the size and shape of individual brain regions.

A recent diffusion MRI study using graph theoretical analysis found that post-chemotherapy (within 6 months) breast cancer survivors demonstrated higher characteristic path length than healthy controls (24). In contrast, our graph theoretical analysis study demonstrated no significant difference in breast cancer survivors, 3–12 months after the completion of chemotherapy. We inferred that it is related to the “psychological resilience” developed after the acute stage of chemotherapy-related neurotoxicity. Some studies have discussed the psychological resilience in patients with breast cancer (82, 83). In McDonald’s fMRI study, significant frontal lobe hyperactivation to support working memory was found in patients with breast cancer. Superimposed on this background, patients showed decreased frontal activation at 1 month after the completion of chemotherapy, with partial return to the previously abnormal baseline at 1 year later (48). Another fMRI study found that women with breast cancer woman had decreased functional connectivity 1 month after chemotherapy, which partially returned to baseline at 1 year in the dorsal attention network (84). The findings of the above studies support our results. Furthermore, in the present study, each group of women demonstrated connectomes with the small-world properties of complex networks rather than those of random networks. Small-world network has high local and global efficiency; therefore, the brain network can effectively transmit information. The human brain has been demonstrated to possess connectomes with small-world properties that not only have the ability to segregate and integrate information but also demonstrate low energy consumption and high efficiency while transmitting and processing information (85).

### Limitations

There are several limitations in this study. There was variability in breast cancer stage, breast cancer subtypes, hormonal treatment and menopause status, which may affect the results of the study. The correlations should be considered with caution, and future research involving a larger sample size of patients with breast cancer is recommended. Second, the cross-sectional design did not allow us to observe the effects of both chemotherapy and psychological stress at different time points in participants with breast cancer. Hence, longitudinal studies are needed to examine these effects.

## Conclusion

This study demonstrated the impact of both chemotherapy and cancer-related psychiatric comorbidity on brain microstructural alterations and cognitive dysfunction in breast cancer survivors. Our study used advanced GQI for graphical theoretical analysis to investigate brain structural alterations in cancer-related trauma survivors. Our findings suggest that both chemotherapy and cancer-related mental health problems may contribute to the development of white matter alterations and cognitive dysfunction in breast cancer survivors, and that these effects may be related to changes in brain network organization. Further studies on this issue with larger samples and longitudinal designs are required to determine the long-term effects of altered brain network organization.

## Data availability statement

The datasets presented in this article are not readily available because of the licenses/restrictions of Chang Gung Memorial Hospital, Chiayi, Taiwan. Requests to access the datasets should be directed to jcweng@mail.cgu.edu.tw.

## Ethics statement

The studies involving human participants were reviewed and approved by the Institutional Review Board of Chang Gung Memorial Hospital, Chiayi, Taiwan (No. 104-5082B, 201700256B0, and 201702027B0). The patients/participants provided their written informed consent to participate in this study.

## Author contributions

All authors listed have made a substantial, direct, and intellectual contribution to the work and approved it for publication.

## Funding

This study was supported by research grants MOST107-2221-E-182-054-MY3 and NSTC111-2221-E-182-021 from the National Science and Technology Council, Taipei, Taiwan, respectively. This study was also supported by grants NMRPD1H0101 ~ 3 from Chang Gung University, Taoyuan, Taiwan and CORPG6G0101 ~ 3 and CORPG6G0121 ~ 3 from Chang Gung Memorial Hospital, Chiayi, Taiwan.

## Conflict of interest

The authors declare that the research was conducted in the absence of any commercial or financial relationships that could be construed as a potential conflict of interest.

## Publisher’s note

All claims expressed in this article are solely those of the authors and do not necessarily represent those of their affiliated organizations, or those of the publisher, the editors and the reviewers. Any product that may be evaluated in this article, or claim that may be made by its manufacturer, is not guaranteed or endorsed by the publisher.

## Footnotes

^1^ http://www.alivelearn.net/xjview
