# Supplementary material for: Detecting microstructural alterations of cerebral white matter associated with breast cancer and chemotherapy revealed by generalized q-sampling MRI
Source: Front Psychiatry. 2023 Jun 9;14:1161246. doi: 10.3389/fpsyt.2023.1161246 (PMC10289548; doi:10.3389/fpsyt.2023.1161246)
Supplement: Supplementary file 1 [file Table_1.DOCX]

**Table S1** Summary of voxel-based between group differences in cross-sectional study

| **Region** | **Figure** | **MNI coordinates (x, y, z)** |
| --- | --- | --- |
| ***ANCOVA*** |  |  |
| corpus callosum (CC) | Fig. 1a, e | 6, 20, 10 |
| left posterior cingulate gyrus (PCG) | Fig. 1b | -8, -34, 34 |
| left middle frontal gyrus (MFG) | Fig. 1c, h | -30, 20 ,40 |
| right superior parietal gyrus (SPG) | Fig. 1d | 28, -66, 50 |
| right tapetum | Fig. 1f | 28, -42, 12 |
| bilateral middle frontal gyrus, orbital (ORBmidF) | Fig. 1g | ±18, 42, -8 |
| left inferior longitudinal fasciculus (ILF) | Fig. 1i | -36, -26, -4 |
| bilateral middle occipital gyrus (MOG) | Fig. 1j | ±36, -76, 26 |
| ***t-test (HC>BB)*** |  |  |
| left middle frontal gyrus (MFG) | Fig. 2a, b | -30, 20, 40 |
| right superior parietal gyrus (SPG) | Fig. 2c, d | 28, -66, 50 |
| bilateral middle occipital gyrus (MOG) | Fig. 2e, f | ±36, -76, 26 |
| ***t-test (HC>BA)*** |  |  |
| corpus callosum (CC) | Fig. 3a | ±8, 12, 24 |
| left middle frontal gyrus (MFG) | Fig. 3b | -30, 22, 38 |
| ***t-test (BB>BA)*** |  |  |
| left posterior cingulate gyrus (PCG) | Fig. 3c | -10, -32, 36 |
| left middle frontal gyrus (MFG) | Fig. 3d | -30, 22, 38 |
| Results in voxel-based analysis were presented in coordinate of Montreal Neurological Institute (MNI) space. | | |
